# The Gut Microbiota and Its Relevance to Peripheral Lymphocyte Subpopulations and Cytokines in Patients with Rheumatoid Arthritis

**DOI:** 10.1155/2021/6665563

**Published:** 2021-01-08

**Authors:** Yuan Li, Sheng-Xiao Zhang, Xu-Fang Yin, Ming-Xing Zhang, Jun Qiao, Xiao-Hong Xin, Min-Jing Chang, Chong Gao, Ya-Feng Li, Xiao-Feng Li

**Affiliations:** ^1^Department of Rheumatology, The Second Hospital of Shanxi Medical University, Shanxi, China; ^2^Key laboratory of Cellular Physiology at Shanxi Medical University, Ministry of Education, China; ^3^Department of Nephrology, Precision Medicine Center, The Shanxi Provincial People's Hospital, Shanxi Medical University, Taiyuan, China; ^4^Department of Pathology, Brigham and Women's Hospital, Harvard Medical School, Boston, MA, USA

## Abstract

Growing experimental and clinical evidence suggests that a chronic inflammatory response induced by gut microbiome critically contribute to the development of rheumatoid arthritis (RA). Previous studies demonstrated the disturbance of lymphocyte subpopulations in RA patients. The purpose of this study was to explore the characteristics of gut microbiome and the associations between bacterium and lymphocyte subpopulations as well as cytokines in patients with RA. Fecal samples from 205 RA patients and 199 healthy controls (HCs) were collected for bacterial DNA extraction and 16S ribosomal RNA (rRNA) gene sequencing. The levels of peripheral lymphocyte subpopulation such as T, B, CD4^+^T, CD8^+^T, NK, T helper 1 (Th1), Th2, Th17, and regulatory T cells (Tregs) of these subjects were detected by flow cytometry combined with standard absolute counting beads. The serum levels of cytokines interleukin-2 (IL-2), IL-4, IL-6, IL-10, IL-17, tumour necrosis factor-*α* (TNF-*α*), and interferon-*γ* (INF-*γ*) were tested by flow cytometric bead array (CBA). Alpha and beta diversity of gut microbiome were explored by bioinformatics analysis. Spearman rank correlation test was used to explore the relationships between gut microbiome and lymphocyte subsets as well as serum cytokines. The diversity and relative abundance of intestinal microbiota in patients with RA were significantly different from those in HCs. Detailly, the abundant of phylum *Proteobacteria* in RA patients was more than that in HCs, while *Firmicutes* was less than in HCs. There was increased relative abundance of genus *Clostridium_XlVa* as well as genus *Blautia*, more abundance of *Ruminococcus2* in patients with lower levels of T, B, CD4^+^T, and Tregs. In addition, the relative abundances of *Pelagibacterium*, *Oxalobacter*, *ClostridiumXlVb*, and *ClostridiumXVIII* were correlated with cytokines. Gut microbiome of RA patients was clearly different from that of HCs. Abnormal bacteria communities are associated with the altered levels of lymphocyte subpopulation and cytokines, which might be one of the pathogenesis of RA.

## 1. Introduction

Rheumatoid arthritis (RA) is a systemic autoimmune disorder characterized by a chronic immune response that leads to inflammation and destruction of synovial joints [1]. The etiological mechanisms involved are heredity, infection, and environmental trigger [2, 3]. Accumulating evidences proposed that gut microbiota was an indispensable environmental factor in the progression of RA [4–8]. Gut microbiota, a major source of microbes, plays a key role in human body defence system [9, 10]. Aberrant immune response was closely associated with dysbiosis of the gut microbiota [7, 8, 11, 12]. Dysbiosis of gut microbiota triggers several types of autoimmune diseases by disturbing the balance of lymphocyte subpopulations such as T helper 1 (Th1), Th2, Th17, and regulatory T cells (Tregs) [5].

Our previous studies have demonstrated that patients with autoimmune disease have a disturbance of lymphocyte subpopulations mainly manifests as insufficient absolute counts of circulating CD4^+^T lymphocyte subsets such as Tregs [13], which was related to disease activities and could be reversed by immunomodulatory drugs (IMiDs) such as low-dose interleukin-2 and sirolimus [14, 15]. However, there is no conclusive evidence to reveal a causal relationship between microorganism and lymphocyte subpopulations with RA.

In this study, we aimed to outline the picture of intestinal microbiota of RA and further to explore the associations between bacterium and lymphocyte subpopulations as well as cytokines.

## 2. Materials and Methods

### 2.1. Study Participants

A total of 404 participants were recruited in this study, comprising 205 RA patients and 199 age and sex-matched healthy controls (HCs). Patients with RA diagnosed in the Second Hospital of Shanxi Medical University were recruited between December 2018 and August 2019. All the patients met the 2010 American College of Rheumatology (ACR)/European League Against Rheumatism (EULAR) classification criteria for RA [16]. HCs were enrolled from the health examination centre of Shanxi Provincial People's Hospital. In addition, the participants who received antibiotics treatment within two months or having a known history of gastrointestinal tract disorders were excluded. Each participant provided informed consent, and the study was approved by the institutional ethics committee of the Second Affiliated Hospital of Shanxi Medical University (Ethics Number: 2019-YX-107).

### 2.2. Sample Collection

All fresh fecal samples collected from participants within a sterile box were transported to the laboratory immediately and then stored at -80°C. Among these patients, 143 of them donated their peripheral blood to test for lymphocytes, and 112 of them were agreed to test cytokine analysis. The bloods' samples were centrifuged (3000 g for 20 min) within one hour of collection. After centrifugation, the plasma was used for lymphocyte subpopulation analysis and the serum for cytokine analysis.

### 2.3. DNA Extraction and Illumina Sequencing

Microbial genome was extracted from approximately 250 mg fecal samples using QIAamp PowerFecal DNA Kit (Qiagen) according to the manufacturer's instructions. The quality of sample DNA was examined by using an agarose gel electrophoresis and NanoDrop One (Thermo Fisher Scientific). DNA extracts were employed for the amplification of the V3–V4 hypervariable regions of the microbial 16S rRNA gene, with KAPA HiFi HotStart Ready Mix (Roche). The products were purified and recovered by FC magic beans Kit (enlighten). Qubit 4.0 (Thermo Fisher Scientific) was used to quantify the purified products. Each sample was diluted to 4 nM. Mix equal volume of each sample for pooling followed by denaturing with NaOH, at least 5% of the Phix library, was added to balance the library polymorphism. Each sample was sequenced on Miseq PE300 (Illumina).

### 2.4. Bioinformatics Analysis

After filtrating and merging the raw data, UPARSE was used to select operational taxonomic units (OTUs) with a similarity cutoff of 97% [17]. Alpha diversity was analyzed by Mothur. Beta diversity was analyzed by QIIME software package. Linear discriminant analysis (LDA) effect size (LEfSe) was analyzed to identify the community differences between groups and distinguish the biomarker species.

### 2.5. Assessment of Lymphocyte Subpopulations and Cytokine in Serum

A modified method of flow cytometry was used to detect the plasma levels of lymphocyte subpopulations 14. The absolute counts and proportions of lymphocyte subpopulations including T (CD3^+^CD45^+^), B (CD19^+^CD45^+^), CD4^+^T (CD3^+^CD4^+^CD45^+^), CD8^+^T (CD3^+^CD8^+^CD45^+^) and NK (CD16^+^CD56^+^CD45^+^), Th1 (CD4^+^IL-2^+^), Th2 (CD4^+^IL-4^+^), Th17 (CD4^+^IL-17^+^), and Tregs (CD4^+^CD25^+^FoxP3^+^). The flow cytometric bead array (CBA) was used to detect the serum levels of IL-2, IL-4, IL-6, IL-10, IL-17, TNF-*α*, and INF-*γ*. Every procedure followed the manufacturer's instructions.

### 2.6. Statistical Analysis

The SPSS 22.0 statistical software was used for analysis. Alpha diversity was compared between groups using nonparametric tests (Mann-Whitney or Kruskal-Wallis); beta diversity was explored using permutational multivariate analysis of variance (PERMANOVA). Data were expressed as mean ± standard or mean ± standard error of mean. Group means were compared using the Mann-Whitney *U* test and Student's *t*-test. Spearman rank correlation test was used for correlation analysis. *P* < 0.05, the difference was statistically significant. *P* < 0.01, the difference was significant.

## 3. Results and Discussion

### 3.1. Clinical Characteristics of Participants

The clinical information and basic characteristics of participants are showed in Table 1. There was no statistically significant difference in age or gender between these two groups (*P* > 0.05).

### 3.2. Gut Microbiota of RA Patients Differed from HCs

All samples were sequenced by high-throughput sequencing method. 5594217 high-quality reads which used to construct OTUs were obtained after filtrating and merging from these samples. Amplicons were clustered into 1051 OTUs at 97% similarity. The rarefaction curves tended to be close to saturated platform, indicating the number of samples was ample. Similar results in the Shannon-Wiener curves and Rank-Abundance curves, which indicated the amount of sequencing data, were abundant enough to reflect most of the microbial species information in the sample.

**Figure 1:**
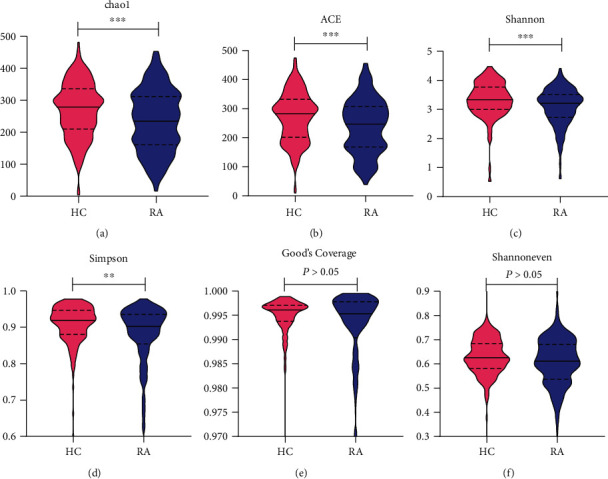
The *α* diversity of the gut microbiomes for the RA patients and the healthy controls has obvious differences (^∗∗∗^*P* < 0.001, ^∗∗^*P* < 0.01).

To determine alpha diversity, Chao1 and ACE index were used to calculate community richness. Shannon and Simpson index were able to evaluate community diversity. Gut microbiota alpha diversity assessed by the number of the observed genera significantly differed between RA and HCs. Compared with HCs, the richness and diversity of gut microbiota in RA was significantly decreased (*P* < 0.01, Figure 1). No significant difference was apparent in Good's Coverage index between HCs and RA (98.99% versus 98.59, *P* > 0.05, Figure 1(e)). The higher the Good's Coverage index, the higher the probability of species being measured in the sample. There was no difference in Shannoneven index between the two groups (*P* > 0.05, Figure 1(f)).

**Figure 2:**
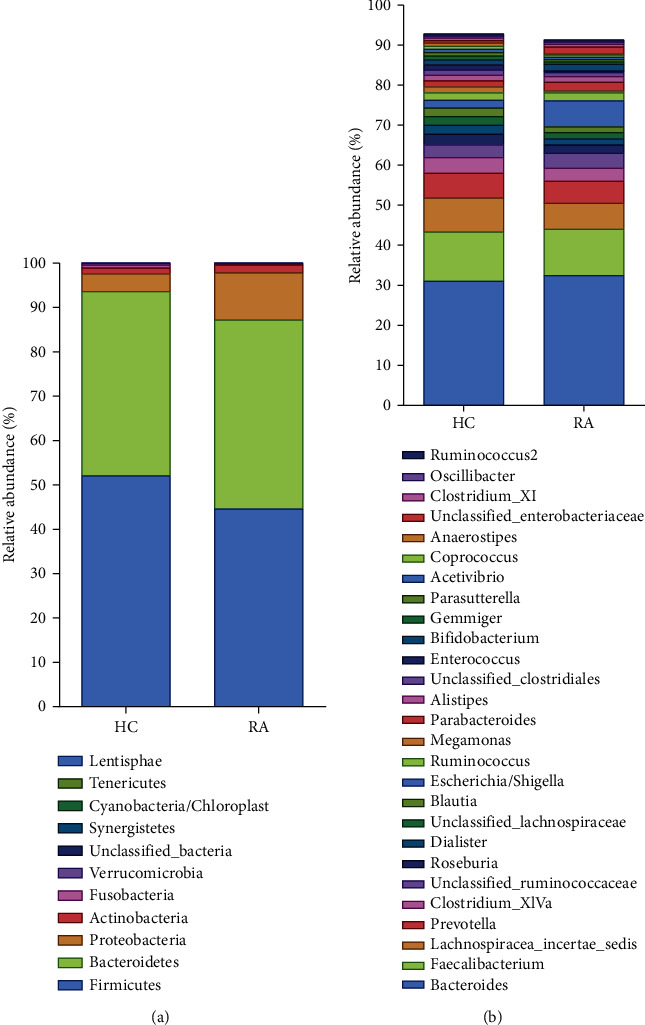
Microbiota composition of healthy controls and RA patients at the phylum (a) and genus (b) levels.

**Figure 3:**
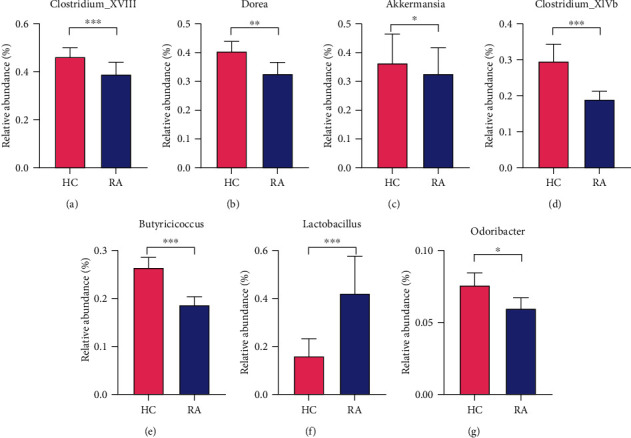
At the generic level, the relative abundances of some gut microbiome in healthy controls and RA patients were compared. Data were expressed as mean ± standard error of mean (^∗∗∗^*P* < 0.001, ^∗∗^*P* < 0.01, ^∗^*P* < 0.05).

For community structure, a relative abundance at the phyla level and a relative abundance larger than 0.5% at the genus level were assessed. Compared with the HCs, the relative abundance of Proteobacteria at phyla level increased in RA, while the relative abundance of Firmicutes declined statistically (*P* < 0.05) (Figure 2(a)). At the genus level, patients with RA had an increased relative abundance of Escherichia/Shigella as well as Ruminococcus2 in RA (*P* < 0.05). However, the relative abundance of *Lachnospiracea_incertae_sedis*, *Prevotella*, *Clostridium_XlVa*, *Roseburia*, *Dialister*, *Unclassified_Lachnospiraceae*, *Blautia*, *Megamonas*, *Unclassified_Clostridiales*, *Gemmiger*, *Parasutterella*, *Acetivibrio*, *Coprococcus*, and *Anaerostipes* were declined in RA (*P* < 0.05) (Figure 2(b)). Noticeably, there were also significant differences in the relative abundance of *Clostridium_XVIII*, *Clostridium_XlVb*, *Lactobacillus*, and so on between the two groups (*P* < 0.05) (Figure 3).

The Bray-Curtis distance was used to calculate beta diversity. PERMANOVA also demonstrated that the microbiota composition significantly differed between the two groups (*P* < 0.01).

### 3.3. Alteration in Gut Microbiota between RA and HCs

**Figure 4:**
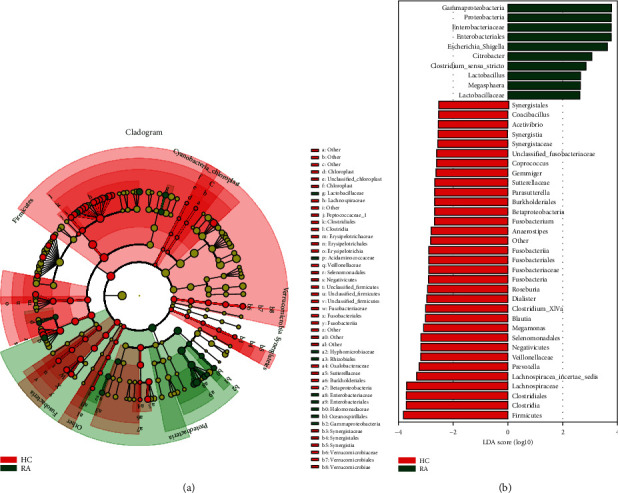
(a, b) LefSe analysis was performed to identify differentially abundant taxa, which are highlighted on the phylogenetic tree in cladogram format (a) and for which the LDA scores are shown (b).

Based on the results of species-abundance comparison between two groups of gut microbiota, the community differences between groups were analyzed from the level of phylum to genus classification by using linear discriminant analysis effect size (LEfSe) [18] (Figure 4). Our results suggested that the relative abundance of the phylum *Proteobacteria*, along with its three families, *Hyphomicrobiaceae*, *Enterobacteriaceae*, and *Halomonadaceae*, were increased in RA patients compared with HCs (*P* < 0.05). In HCs, phylum *Firmicutes* had the largest LDA score.

### 3.4. The Relevance between Different Gut Microbial Genera and Peripheral Lymphocyte Subpopulations

Considering the correlation between intestinal microorganisms and peripheral blood (PB) lymphocyte subpopulations, taxa that showed significant difference in relative abundance were selected to evaluate the relevance between gut microbiota composition and lymphocyte subpopulations.

**Figure 5:**
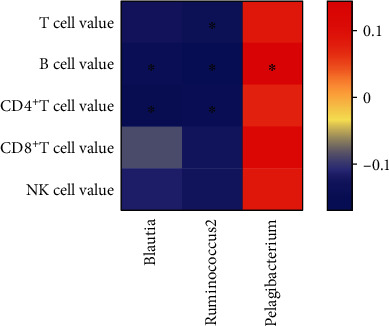
A heat map shows the correlation between different gut microbial species and lymphocyte subpopulations. Colors indicate the Spearman rank correlation (^∗^*P* < 0.05).

Spearman correlation coefficients were computed between each bacterial taxa and each of the plasma levels of T, B, CD4^+^T, CD8^+^T, and NK cells. The relative abundance of *Blautia*, *Ruminococcus2*, and *Odoribacter* showed significant negative correlations with these markers (*P* < 0.05). Detailly, *Blautia* was related to the absolute numbers of B and CD4^+^T cells (*P* < 0.05). *Ruminococcus2* was related to the absolute numbers of T, B, and CD4^+^T cells (*P* < 0.05). Besides, the relative abundance of *Pelagibacterium* was positively correlated with the absolute numbers of B cells (*P* < 0.05) (Figure 5).

**Figure 6:**
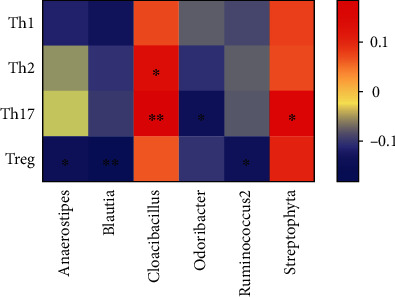
A heat map shows the correlation between different gut microbial species and CD4^+^T lymphocyte subpopulations. Colors indicate the Spearman rank correlation (^∗∗^*P* < 0.01, ^∗^*P* < 0.05).

As for CD4^+^T cell subsets such as Th1, Th2, Th17, and Tregs, the relative abundances of *Anaerostipes*, *Blautia*, *Odoribacter*, and *Ruminococcus2* were significantly and negatively correlated with these markers (*P* < 0.05). Additionally, the relative abundance of *Cloacibacillus* was positively correlated with the absolute numbers of Th2 (*P* < 0.05) and Th17 (*P* < 0.01), and the relative abundance of *Streptophyta* showed a positive correlation with the absolute numbers of Th17 (*P* < 0.05) (Figure 6).

### 3.5. The Relevance between Different Gut Microbial Genera and Cytokines

**Figure 7:**
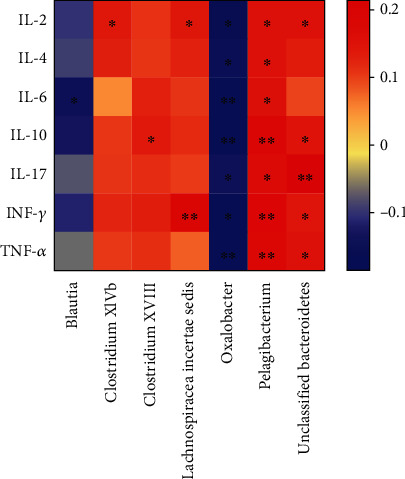
A heat map shows the correlation between different gut microbial species and cytokines. Colors indicate the Spearman rank correlation (^∗∗^unadjusted *P* < 0.01, ^∗^*P* < 0.05).

Based on the results that analyzed by LEfSe, taxa that showed significant difference in relative abundance were selected to evaluate the relevance between gut microbiota composition and cytokines. Spearman correlation coefficients were computed between each bacterial taxa and each of the serum levels of IL-2, IL-4, IL-6, IL-10, IL-17, TNF-*α*, and INF-*γ*. As showed in Figure 7, the relative abundance of *Pelagibacterium* had a significant positive correlation with these cytokines (*P* < 0.05). On the contrary, the relative abundance of *Oxalobacter* was negatively correlated with these cytokines (*P* < 0.05). There was less abundance of *Unclassified-Bacteroidetes* in patients with lower levels of IL-2 (*P* < 0.05), IL-10 (*P* < 0.05), IL-17 (*P* < 0.01), INF-*γ* (*P* < 0.05), and TNF-*α* (*P* < 0.05). The relative abundances of *ClostridiumXlVb* and *ClostridiumXVIII* are positively correlated with IL-2 and IL-10, respectively (*P* < 0.05). These findings indicated different gut microbiota closely associated with serum cytokines in RA.

## 4. Discussion

The microbiome components are an attractive source of antigens capable of inducing RA and have been the most investigated as potential causal agents [19]. Consistent with previous studies [20–22], our findings identified gut microbiome alteration in individuals with RA by both alpha diversity and beta diversity.

Specific gut microbiota played an important role in the pathogenesis of RA. In our research, phylum *Proteobacteria*, the largest phylum of bacteria, including many pathogenic bacteria, such as *Enterobacter* and *Salmonella*, were more abundant in RA patients. These pathogens may invade the intestinal mucosa to increase its permeability, the proliferation of inflammatory cells, and secretion of inflammatory mediators of them, leading to immune inflammatory response. At the genus level, patients with RA displayed a mean 3-fold overabundance of *Lactobacillu*, which characterized a potentially harmful microorganisms and participated in the development of inflammatory arthritis [23]. Further, *Lactobacillu* was successfully used to induce arthritis model because *Lactobacillu* might result in an imbalance of Th17/Tregs homeostasis by mediating TLR2–TLR4 signaling [24]. In addition, some studies have also shown that *Lactobacillus casei* (L. *casei*) supplementation has an active improvement for patients with RA [25, 26], suggesting different species of Lactobacillus have different effects on arthritis, and further detailed research is needed.

We found insufficient abundance of *Clostridium_XlVa*, *Clostridium_XlVb*, and *Clostridium_XVIII* in patients with RA. Interestingly, intestinal *Clostridia* species was reported to alleviate inflammatory response by produce IL-10 through specific capsule component polysaccharide A (PSA) and induce Treg proliferation [27]. *Clostridium_XlVa* has anti-inflammatory properties by enhancing transforming growth factor-beta (TGF-*β*) expression and inducting or accumulating Tregs [27–29]. Research shows that Treg can maintain tolerance to autoantigens and eliminate autoimmunity and play an important role in maintaining immune tolerance to dietary antigens and intestinal flora [30]. The decrease in the number and function of Treg cells leads to the deficiency of immune tolerance and abnormal immune response to autoantigens, which are involved in the occurrence and development of RA [31]. As Tregs play a significant role in anti-inflammation and maintaining immune system homeostasis [32], insufficiency of these floras might attenuate Tregs promoting the deficiency of immune tolerance.

To data, the existing research findings about *Prevotella* are controversial. Vaahtovuo et al. [11] found that the relative abundance of *Prevotella* in the intestine of RA patients decreased, which was consistent with our findings. But *Prevotella copri* increased among new-onset untreated RA patients [8, 33]. Also, Rodrigues et al. [34] found an increase in relative expression units (REU) of *Prevotella* species in stool samples from Brazilian RA patients. In fact, for the existing disputes, further research and exploration are needed. It is confirmed that Escherichia play a role in the pathogenesis of arthritis in germ-free arthritis-prone rats [35], which is consistent with our results that showed an increase in the relative abundance of Escherichia in patients with RA.

Immunopathogenesis of RA is mainly manifested in immune system dysregulation which is characterized by the presence of autoantibodies and autoreactive T cells. It was found that the function of circulating Treg cells in plasma and synovium is impaired [36], and Th17 cell abundance increased [37, 38]. The imbalance of Th17/Treg is the main factor for the abnormality of autoreactive T cells. Presumably, the imbalance of intestinal flora in susceptible hosts may destroy the balance of Th17/Treg [39], which leads to the destruction of immune tolerance, following by a systemic immune disequilibrium. This immunologic derangement favors proinflammatory responses resulting in joints and other tissue damage. Our results showed that the relative abundance of *Ruminococcus2* increased in RA patients and negatively correlated with the absolute number of T, B, CD4^+^T, and Tregs, while the relative abundance of *Blautia* was decreased and negatively correlated with B and CD4^+^T cell. The correlation analysis between intestinal microorganisms and lymphocyte suggests that alteration of gut microbiota might play a role in the pathogenesis of RA by modulating the immune systems of human.

Cytokines played an important role in the occurrence of inflammation and the pathogenesis of autoimmune diseases [40, 41]. TNF-*α* plays a fundamental role through activation of cytokine and chemokine expression, expression of endothelial-cell adhesion molecules, protection of synovial fibroblasts, promotion of angiogenesis, suppression of regulatory T cells, and induction of pain [2, 42, 43]. *Pelagibacterium*, *Oxalobacter*, and *Blautia* were related to TNF-*α*. IL-6 and IL-17 are closely related to the bone erosion by enhancing the differentiation and activation of osteoclast [44]. In our study, the relative abundance of *Pelagibacterium* was increased while *Oxalobacter* and *Blautia* decreased. Just as we expected, *Pelagibacterium* showed a significant positive correlation with these cytokines; *Oxalobacter* and *Blautia* were negatively correlated with these cytokines, suggesting intestinal microflora might participate in pathogenic progress by altering levels of cytokines.

## 5. Conclusions

Richness and diversity of intestinal flora in RA patients were impaired, which might participate in the pathogenesis of RA by modulating the immune systems with lymphocyte subpopulations and cytokines. The discovery of the associated intestinal microbiota of RA may provide a new idea for RA treatment.

## Figures and Tables

**Table 1:** Age and gender information of the enrolled participants.

Group	Number	Sex		Age
		Male	Female	
HC	199	81	118	50.03 ± 11.04
RA	205	75	130	52.11 ± 12.24
*P*		0.395		0.074

## Data Availability

The SRA data used to support the findings of this study are available from the corresponding author.
